# Sustainable oil palm trunk fibre based activated carbon for the adsorption of methylene blue

**DOI:** 10.1038/s41598-023-49079-0

**Published:** 2023-12-13

**Authors:** Muniandy Gayathiri, Thiruchelvi Pulingam, K. T. Lee, Azam Taufik Mohd Din, Akihiko Kosugi, Kumar Sudesh

**Affiliations:** 1 School of Biological Sciences, Universiti Sains Malaysia, 11800 Penang, Malaysia; 2 School of Chemical Engineering, Engineering Campus, Universiti Sains Malaysia, Seri Ampangan, 14300 Nibong Tebal, Penang Malaysia; 3 Japan International Research Center for Agricultural Sciences (JIRCAS), Biological Resources and Post-Harvest Division, 1-1 Ohwashi, Tsukuba, Ibaraki 305-8686 Japan; 4 University of Tsukuba, 1-1-1 Tennodai, Tsukuba, Ibaraki 305-8577 Japan

**Keywords:** Biochemistry, Environmental sciences, Chemistry

## Abstract

Activated carbon (AC) is becoming the limelight due to its widespread application as an adsorbent for wastewater treatment, gases, and catalysis. However, its high consumption and price have drawn more attention to the sustainable use of natural resources as precursor for AC production. This study focuses on synthesising AC from two types of oil palm trunk (OPT) fibres, a significant agricultural waste products produced by Malaysia's thriving palm oil industries. The BET surface area of about 2057.9 m^2^ g^−1^ was achieved by chemical activation with phosphoric acid (H_3_PO_4_). The efficiency of the synthesised AC was critically analysed based on the adsorption experiments with methylene blue (MB) by varying several parameters (dosage of adsorbent, pH, initial dye concentration, and temperature of the solution) to elucidate the adsorption mechanism(s). A maximum adsorption capacity of 320.4 mg g^−1^ at 50 °C was achieved, and the Temkin (r^2^ = 0.98, 0.95, 0.95) and Langmuir (r^2^ = 0.94, 0.93, 0.95) isotherm models fitted the adsorption process better than the Freundlich (r^2^ = 0.95, 0.90, 0.86) model. Besides, the pseudo-second-order model (r^2^ > 0.90) best described the adsorption process, favouring chemisorption over physisorption. Thermodynamics showed MB adsorption on AC was spontaneous except at the highest dye concentration. It was exothermic at lower dye concentrations (50 and 100 mg L^−1^) and endothermic at higher ones (300, 500, and 700 mg L^−1^). In a nutshell, this study reveals that OPT fibre is a promising precursor for synthesising highly porous AC for the adsorption of MB dye.

## Introduction

The manufacturing and consumption of dye in various forms in our daily life generate vast amounts of dye wastewater, primarily from industries such as papermaking, textile, food, printing, cosmetics, etc. Approximately 7 × 10^7^ tons of synthetic dyes have been produced to date globally, with the textile industry contributing over 10,000 tons^1^. One of the common synthetic dyes used in the textile industry is the cationic MB dye which is also discharged to the water surface in large amounts due to extensive industrial usage^2^. MB is toxic, carcinogenic, and non-biodegradable; it poses a threat to human health and the environment^3^.

Numerous methods for MB and other synthetic dye removals from wastewater have been investigated, such as membrane filtration, advanced oxidation, flocculation, biosorption, liquid–liquid extraction, etc. However, adsorption was found to have numerous advantages as it requires less space than the biological system, flexible to design and operate, can remove organic compounds, and is robust to chemicals^4^. AC is commonly used in adsorption as it is a porous material, rich with carbon, surface area, pore volume, and tunable pore size, and has good stability for temperature and chemicals^5^.

Various agricultural precursors were used to synthesise AC, such as bamboo Hameed et al.^6^, corncob Feng et al.^7^, sunflower seed Abbas and Ahmed^8^, sugarcane bagasse Akl et al.^9^, and flax fibre Williams and Reed^10^. In a prior investigation, the Zn(OH)_2_-AC composite, synthesised from coffee waste, exhibited a peak adsorption capacity of 303.0 mg g^−1^ at 318 K and pH 7 when used to eliminate malachite green^11^. Besides, it was found in a previous literature that the optimal conditions for achieving maximum MB removal of 163.60 mg g^−1^ involve using 0.06 g of mangosteen peels activated carbon, maintaining a pH of 6 in the MB solution, and allowing a contact time of 20 min^12^. Moreover, a past study demonstrated that enhancing the activated carbon (AC) derived from acorn shells through ZnCl_2_ activation with magnetic properties resulted in an impressive adsorption capacity of 357.1 mg g^−1^ at 298 K^13^.

This study used different types of fibre from the OPTs to produce the AC. Oil palm is a valuable commercial cash crop, and Malaysia is the second largest palm oil producer globally after Indonesia^14^. However, the oil palm plantation and industry contribute to more than 70 million tons of lignocellulosic biomass wastes^15^. To date, there has been a significant increase in global concern about recycling oil palm biomass to provide sustainable resources. Besides, it was stated in previous literature that leaving diseased OPTs in the plantation after replanting activity can cause early deaths among young palms^16^. OPT is a monocotyledon with non-wood structure made up of parenchyma and vascular bundle fibre^17^. The parenchyma tissue stores food whereas the vascular bundle tissue provides mechanical support to the tree^18^.

The precursors in this work were chemically impregnated with H_3_PO_4_ as it requires lower temperature during activation, produces high carbon yield, and contributes large pores^16^. The synthesised ACs were characterised for surface morphology and adsorption experiments were conducted with different parameters (AC dosage, pH, initial dye concentration and temperature of the solution). The results obtained from the adsorption capacity and percentage of dye removal were fitted into different adsorption models to determine the adsorption mechanisms involved. However, further improvisions can be done to this study such as varying the carbonisation conditions, adsorption experiment with real wastewater and reusability study of the synthesised AC. This work offers a comprehensive analysis of the physicochemical factors that contribute to the adsorption mechanisms of vascular bundle and parenchyma-based adsorbents in their ability to remove MB dye. The objective of this study is to provide an in-depth knowledge of the dye adsorption process by providing extensive insights into the performance of these adsorbents. To the best of our knowledge, this is the first study that synthesizes AC using the vascular bundle and parenchyma fibre independently.

## Materials and methods

### Materials

MB dye (λ_max_ = 664 nm) with molecular weight of 373.9 g mol^−1^ was purchased from Sigma-Aldrich (M) Sdn Bhd, Malaysia and used as the synthetic dye (adsorbate) in this study. The MB standard curve of absorbance versus concentration was plotted and was used to determine the remaining dye concentrations for the adsorption experiment at different time intervals. Phosphoric acid of 85% was purchased from QRec. Permissions to collect the plant specimens (OPT fibre) was granted by the SATREPS Kluang pilot plant, Johor.

### Preparation of OPT based ACs by chemical activation

The OPT was chipped, washed in a dipping tank, wet-milled, screw-pressed, and dried before use. The vascular bundle and parenchyma fibres were separated using different sizes of sieve where the vascular bundle was the retentate of the 1 mm mesh whereas parenchyma was the filtrate of the 0.25 mm mesh. The parenchyma and vascular bundle fibres were impregnated in 85% H_3_PO_4_ with an impregnation ratio (acid (g)/fibre (g)) of 0.5, 1.5, 2.5, and 3.5^19^. The solid–liquid mixtures were dried in the oven at 105 °C and carbonised in a horizontal tubular furnace at 600 °C for 1.5 h in an inert atmosphere (N_2_ gas flow rate at 200 cm^3^ min^−1^) with a heating rate of 10 °C min^−1^. Then, the dried samples were weighed before and after carbonisation to determine the percentage yield of AC. The carbonised samples were thoroughly washed using Büchner funnel until the pH of the filtrate water was ~ 7 and dried at 105 °C.

### Characterisation of the lignocellulosic precursors and activated carbon

The precursors were analysed for main elements such as carbon, hydrogen, nitrogen, sulphur, and oxygen based on combustion whereby the ratio of the elements was later measured by gas chromatography based on the products from combustion. The Brunauer–Emmett–Teller (BET) surface area analyser (Micromeritics ASAP 2020, USA), was used to determine the specific surface area, pore volume and pore size of the ACs. Besides, the energy dispersive X-ray spectroscopy (EDX) with FEI Quanta FEG 650 SEM were used to determine the elemental compositions on the surface of the precursors and ACs with high pore surface area.

### Adsorption experiment

MB dye stock solution of 1000 mg L^−1^ was prepared and diluted as desired. A 0.1 g of ACs were added to 100 mL of MB dye solution (100 mg L^−1^) and the adsorption was carried out in an incubator shaker for 12 h at 30 °C and 200 rpm. The pH was maintained within 6–7. The absorbance of the samples was measured using UV–vis spectrophotometer at wavelength of 664 nm at intervals after removing the AC through centrifugation (8000 rpm, 6 min)^20^. The concentration of the sample was determined using the MB standard curve. The adsorption capacity (equilibrium at different time) and percentage removal of dye were calculated based on the Eqs. (1), (2), and (3).

The adsorption capacity at equilibrium, *q*_*e*_:

1 $$q_{e} = \frac{{\left( {{\text{C}}_{0} {-}{\text{C}}_{{\text{e}}} } \right)*V}}{W}$$

where C_0_: initial dye concentration (mg L^−1^), C_e_: equilibrium dye concentration (mg L^−1^), *V*: volume of solution (L), *w*: mass of the AC (g).

The adsorption capacity, *q*_*t*_, at time, *t*:

2 $$q_{t} = \frac{{\left( {{\text{C}}_{0} {-}{\text{C}}_{{\text{e}}} } \right)*V}}{W}$$

where C_0_: liquid phase concentration of the dye at initial (mg L^−1^), C_*t*_: liquid phase concentration of the dye at any time (mg L^−1^), *V*: volume of solution (L), *w*: mass of the AC (g).

The percentage removal of MB:

3 $$\% R = \frac{{\left( {{\text{C}}_{0} {-}{\text{Ct}}} \right)*100}}{{{\text{C}}_{0} }}$$

where C_0_: liquid phase concentration of the dye at initial (mg L^−1^), C_*t*_: liquid phase concentration of the dye at any time (mg L^−1^), *V*: volume of solution (L), *w*: mass of the AC (g).

The effects of different parameters: adsorbent dosage (12.5–50 mg), pH of the solution (3–11), initial dye concentration (50–700 mg L^−1^), and temperature (30–50 °C) on the adsorption capacity of MB dye were determined in this study. The Fourier transform infrared (FTIR) spectroscopy (Perkin Elmer, spectrum 2000, USA) was then used for characterising the surface functional groups of AC before and after adsorption of MB. All methods were carried out in accordance with relevant guidelines^21^.

## Results and discussion

### Characterisation of precursors and activated carbon

Table 1 shows that the parenchyma fibre which is in a soft powdery form whereas the vascular bundle fibre has a hard needle-like structure. The precursors were analysed for elements such as carbon, hydrogen, nitrogen, sulphur, and oxygen. There was no significant difference in the elemental compositions between the parenchyma and vascular bundle fibres as indicated in Table 1. The carbon and oxygen content were approximately 43% and 50%, respectively for both fibres which shows that the OPT biomass may be a suitable precursor to synthesise AC.

**Table 1:** Elemental analysis of parenchyma and vascular bundle fibres.

Samples	Elemental analysis				
	Carbon %	Hydrogen %	Nitrogen %	Sulphur %	Oxygen %
Parenchyma	43.18	4.70	0.74	0.40	50.98
Vascular bundle	43.47	7.15	0.67	0.57	48.14

The results for the BET analysis are as shown in Table 2 where the precursor had a very low BET surface area of 2.5 m^2^ g^−1^ and 1.7 m^2^ g^−1^ for parenchyma and vascular bundle fibre respectively. However, after chemical treatment of H_3_PO_4_/fibre (0.5), there was a drastic increment in the BET surface area up to 593.0 m^2^ g^−1^ (parenchyma) and 941.3 m^2^ g^−1^ (vascular bundle). This may be due to the role of H_3_PO_4_ where it penetrated deeply into the carbon structure of the fibre, forming new pores (mesopores and micropores) that resulted in an increase in the surface area^22^. Similar results were obtained by Zakaria et al.^23^ where the mangrove precursor with surface area of 0.0045 m^2^ g^−1^ increased to 789.2 ​ m^2^ g^−1^ after H_3_PO_4_ chemical activation at 300 °C. The highest surface area was achieved by ACP1.5 and ACVB1.5 with 1599.8 and 2057.9 m^2^ g^−1^ respectively and were further analysed by SEM and adsorption experiments.

**Table 2:** Pore characteristics of untreated fibre, commercial and phosphoric acid treated activated carbon with different impregnation ratios.

Type of sample	Pore characteristics		
	BET surface area (m^2^ g^−1^)	Total pore volume (cm^3^ g^−1^)	Average pore width (nm)
UP	2.5	0	0.9
UVB	1.7	0	0.7
CAC	983.8	0.5	2.0
ACP0.5	593.0	0.3	2.0
ACP1.5	1599.8	1.0	2.6
ACP2.5	1461.7	1.4	3.8
ACP3.5	1333.1	1.6	4.7
ACVB0.5	941.3	0.5	2.2
ACVB1.5	2057.9	1.4	2.6
ACVB2.5	1396.8	1.3	3.6
ACVB3.5	1281.4	1.5	4.7

However, the surface area of the synthesised ACs decreased when the IR was further increased to 2.5 and 3.5. This may be due to the dehydration of H_3_PO_4_ which formed an insulating layer and prevented the activation by H_3_PO_4_ on the OPT fibre which then resulted in the inhibition of internal pore formation^24^. Table 3 shows the surface area obtained for different precursors when H_3_PO_4_ was used as the chemical reagent. The surface area of the AC synthesised depends on the type of precursor and the conditions set during carbonisation (concentration of chemical reagent, activation temperature, and the holding time). Besides, when the carbonisation condition and concentration of the H_3_PO_4_ were similar, the type of precursor affects the BET surface area, 1545.44 m^2^ g^−1^ (eucalyptus branches) whereas 2375 m^2^ g^−1^ (humins) as shown in Table 3^25,26^. Therefore, the type of precursor also contributes to the pore characteristics of the AC.

**Table 3:** Activated carbon production from various biomass precursor via chemical activation with phosphoric acid.

Precursor	Carbonisation conditions			BET surface area (m^2^ g^−1^)	References
	Chemical reagent	Temperature (°C)	Time (h)		
Eucalyptus branches	40 wt% H_3_PO_4_	400	3	1545.44	^26^
Kenaf core fiber	30 wt% H_3_PO_4_	500	1	299.02	^27^
Humins	40 wt% H_3_PO_4_	400	2	2375	^25^
Oil palm fibre	85 wt% H_3_PO_4_	800	2	715.63	^28^
Oil palm trunk (vascular bundle fibre)	85 wt% H_3_PO_4_	600	1.5	2000	This study

The prepared ACs were denoted as ACXY, where AC: activated carbon, X: type of fibre (P: parenchyma and VB: vascular bundle) and Y: IR (0.5/1.5/2.5/3.5). The untreated fibres were denoted as UX (where U: untreated whereas X: type of fibre) and the commercial AC which is of laboratory grade purchased from R&M Chemicals was denoted as CAC.

**Figure 1:**
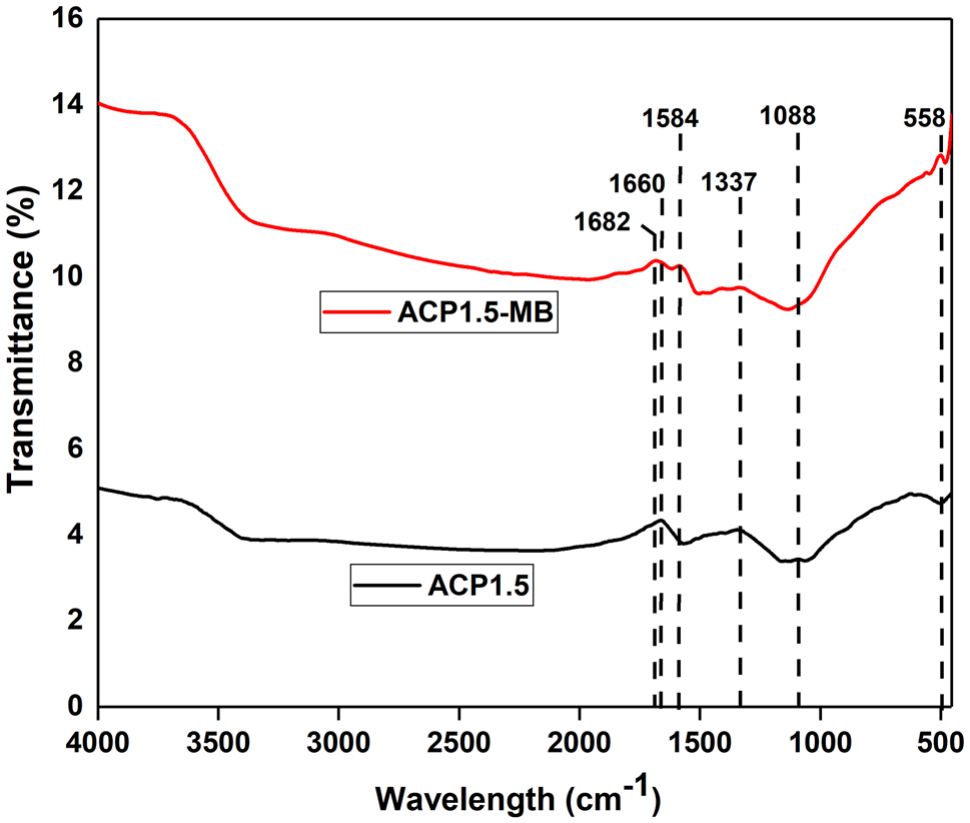
Effect of impregnation ratio (mass of acid/mass of fibre) on the percentage yield and BET surface area of the AC prepared from (**A**) Parenchyma and (**B**) vascular bundle fibre (chemical activation done with phosphoric acid and carbonisation at 600 °C for 1.5 h) and SEM and EDX spectra of parenchyma (**C**) untreated, (**D**) activated carbon and vascular bundle (**E**) untreated, and (**F**) activated carbon [magnification: ×1000; scale bar 70 µm].

The relationship between the yield and the BET surface area of the prepared AC at different IR is as shown in Fig. 1A,B. The increase in the IR from 0.5 to 2.5 increased the yield of AC slightly just as reported by Yorgun et al.^29^ where the yield of AC produced from paulownia wood treated with H_3_PO_4_ increased when IR was increased from 1 to 4. This may be due to the effect of H_3_PO_4_ during chemical impregnation causing the cellulose, hemicellulose, and lignin biopolymers to be redistributed while the aliphatic compounds may have converted to aromatic compounds^30^. On the other hand, the BET surface area increased drastically when the IR was increased from 0.5 to 1.5 and then decreased gradually for the ACs prepared from both types of fibre (Fig. 1A, B). The presence of acid derivatives on the fibre may have increased the rate of pore formation, however, excess acid has the potential to break the lignocellulose bonds extensively and shrink the structure, which subsequently suppresses the surface area of the AC^31^.

The untreated parenchyma fibre presented in Fig. 1C has a variety of randomly distributed circular pores of different sizes whereas the AC from parenchyma fibre with highest surface area was able to form large pores as shown in Fig. 1D. The untreated vascular bundle fibre in Fig. 1E has uniformly distributed silica on the external surface. Similar findings were observed by Davamani et al.^32^ where the surface of empty fruit bunch of oil palm had accretion of silica with a perforated bottom. The white compounds were still found on the surface of vascular bundle-based AC with highest BET surface area (Fig. 1F).

There is an increase in carbon from 53.5 to 79.2% (parenchyma) and from 29.1 to 86.4% (vascular bundle) and a decrease in the oxygen content after chemical activation. This could be due to gasification and release of volatile matter during the activation process^33^. The phosphorus content on the ACs could be due to H_3_PO_4_ impregnation, which formed polyphosphates that retained on the surface of fibres as insoluble metal phosphates or entrapped in the ACs’ pores^34^. The nature of the precursor and the chemical activation method play a pivotal role in the synthesis of AC.

### Adsorption experiment

#### Effect of dosage of AC

**Figure 2:**
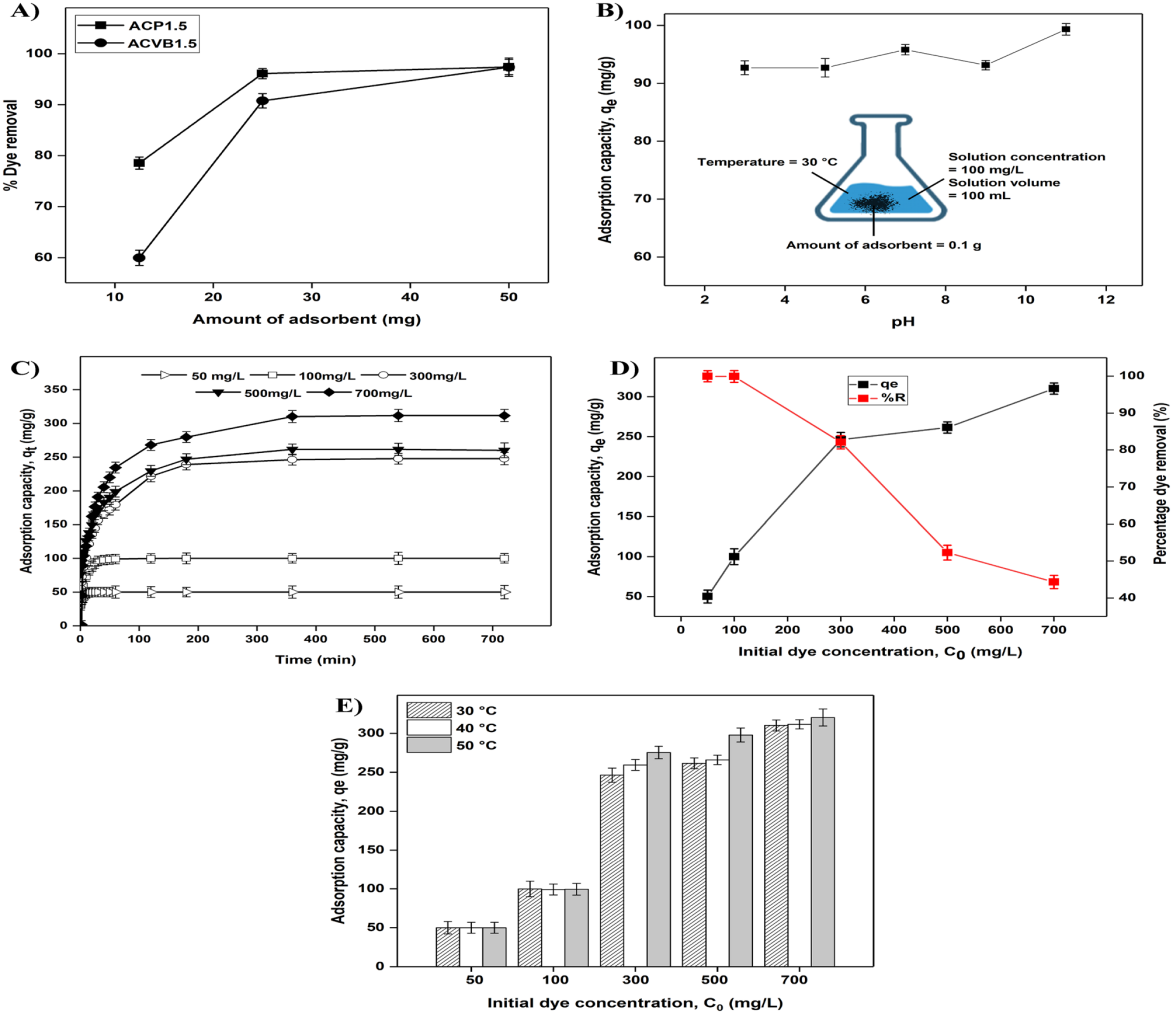
(**A**) Effect of dosage of adsorbent on the percentage of dye removal for activated carbon synthesised from parenchyma (ACP1.5) and vascular bundle (ACVB1.5) fibre, (**B**) Effect of pH on the adsorption capacity of MB dye on the adsorbent, (**C**) Adsorption capacity at different time (*qt*), (**D**) Relationship between adsorption capacity at equilibrium (*q*_*e*_) and percentage dye removal, (**E**) effect of initial dye concentration and temperature on the adsorption capacity of activated carbon.

It can be seen that ACP1.5 showed a better dye removal of 78.5% compared to ACVB1.5 with only 59.8% when the least dosage of AC (12.5 mg) was used for the adsorption of MB dye with concentration of 50 mg L^−1^ (preliminary adsorption) in Fig. 2A). The percentage dye removal increased initially and remained almost similar for both fibres. The adsorption between the MB dye and the AC may have reached saturation at the highest dosage of the adsorbent^35^. The optimum dosage of 50 mg with dye removal of 97% was achieved for both types of ACs in this study. However, in terms of future application of AC for industrial scale, it can be deduced that ACP1.5 would be a better choice than ACVB1.5 for great adsorption of MB with a lesser dosage of AC. The adsorption of ACP1.5 with different parameters was further studied to understand the adsorption process.

#### Effect of pH of the solution

The increase in the pH from 3 to 11 showed an increment in the adsorption capacity from 92.7 to 99.4 mg g^−1^ in Fig. 2B). However, no significant increment was observed, therefore, the synthesised AC is robust to different ranges of pH. The slightly higher adsorption capacity at pH of 11 could be due to the dominance of negative charge on the surface of the AC, which created an electrostatic attraction between the positively charged MB and negatively charged OH^−^ ions from the buffer^36^.

The neutral pH was chosen as the optimum pH for further adsorption experiments and not pH 11 as using great amount of buffer to adjust the basicity of the solution is not economically feasible and environmentally benign.

#### Effect of initial dye concentration and contact time

There is an increasing trend in the maximum adsorption capacity as the initial dye concentration increased as shown in Fig. 2C. The large number of molecules at high initial dye concentration can cause the diffusion rate of the dye molecules through the layer of the surface boundary towards the internal pores on the surface of the AC^37^. The samples with lower initial dye concentrations (50 and 100 mg L^−1^) rapidly attained equilibrium (< 100 min). However, higher initial dye concentrations (300, 500 and 700 mg L^−1^) achieved equilibrium much later (> 360 min). Fewer dye molecules occupy the vacant binding sites rapidly until saturation point compared to when there are more dye molecules with the dosage of adsorbent being the limiting factor.

Figure 2D shows that an increase in the dye concentration induced an increase in the adsorption capacity at equilibrium, q_e_, 50 mg L^−1^ (50.0 mg g^−1^), 100 mg L^−1^ (100.0 mg g^−1^), 300 mg L^−1^ (246.3 mg g^−1^), 500 mg L^−1^ (261.5 mg g^−1^), and 700 mg L^−1^ (310.2 mg g^−1^). Previous literatures obtained a similar trend and this could be due to the availability of large driving force for the MB dye to migrate to the adsorption site on the AC^38–41^. In contrary, the percentage of dye removal (%R) decreases as the initial dye concentration increases, 50 mg L^−1^ (100%), 100 mg L^−1^ (100%), 300 mg L^−1^ (82.1%), 500 mg L^−1^ (52.3%), and 700 mg L^−1^ (44.3%). Al-Ghouti and Al-Absi^42^ reported that increasing the initial MB dye concentration caused the adsorption sites on the AC prepared from olive stones to become saturated which subsequently suppressed the dye removal efficiency which can be a reason for the decrease in percentage dye removal in this study as well.

Biomass from different plants is always used to produce AC for dye adsorption studies due to abundant availability and lignocellulosic properties to aid adsorption. Table 4 shows different types of biomasses used and their adsorption capacity for MB dye adsorption at various initial dye concentrations.

The initial dye concentration and the adsorption capacity for different precursors (Table 4) is random. Therefore, in the adsorption process, other factors may have affected the adsorption capacity in addition to the initial dye concentration. For instance, in a previous literature, it was shown that the atmosphere at which the precursor is activated highly affects the synthesised AC where an atmosphere with oxidant resulted in AC with more oxygen content, higher surface area, and great surface negative charge^43^.

**Table 4:** Adsorption capacity of various biomass derived adsorbents for MB dye of different initial dye concentrations.

Adsorbent	Dye	Initial dye concentration (mg L^−1^)	Adsorption capacity, *q*_*e*_ (mg g^−1^)	Reference(s)
Rubber seed pericarp	Methylene blue	200	415.8	^44^
Sugarcane bagasse waste	Methylene blue	200	122.9	^45^
*Acacia* wood	Methylene blue	300	210.10	^46^
Grass waste	Methylene blue	300	372.2	^47^
*Eucommia ulmoides* Oliver	Methylene blue	100	541.0	^48^
Palm fibres (from sheath)	Methylene blue	500	271.0	^49^
Oil palm empty fruit bunch	Methylene blue	250	75.0	^50^
Mangrove pile leftovers	Methylene blue	600	408.9	^23^
Oil palm trunk fibre (Parenchyma)	Methylene blue	700	320.4	This study

#### Effect of temperature and initial dye concentration 

The effect of temperature on the adsorption capacity (Fig. 2E) shows that the adsorption capacity was unaffected by the temperature for lower initial dye concentrations (50 and 100 mg L^−1^) but it increased with temperature for higher initial dye concentrations (300, 500, and 700 mg L^−1^).

During the adsorption reaction, the increasing amount of adsorbate molecules at higher initial dye concentrations consume energy^42^. Conversely, at lower initial dye concentrations, the small number of dye molecules may have adequate binding sites and may not require energy for the adsorption process.

## Adsorption isotherms, kinetics and thermodynamics

### Adsorption isotherms

Adsorption isotherm parameters for each model, namely, Langmuir, Freundlich and Temkin were calculated based on non-linear fitting method at three different temperatures (30, 40 and 50 °C) to understand the adsorption process. The non-linear model for each isotherm is as below:

Langmuir

4 $$q_{e} = \frac{{q_{m} {\text{K}}_{{\text{L}}} {\text{C}}_{{\text{e}}} }}{{\left[ {{1 } + {\text{ K}}_{{\text{L}}} {\text{C}}_{{\text{e}}} } \right]}}$$

Freundlich

5 $$q_{e} = {\text{K}}_{{\text{F}}} {\text{C}}_{{\text{e}}}^{{{1}/{\text{n}}}}$$

Temkin

6 $$q_{e} = {\text{B}}ln\left( {{\text{K}}_{{\text{T}}} {\text{C}}_{{\text{e}}} } \right)$$

where K_L_: Langmuir constant (L mg^−1^), K_F_: Freundlich constant (mg g^−1^) (L mg^−1^)^1/n^, 1/n: Measure of adsorption intensity, B = RT/b, R = gas constant (8.314 J mol^−1^ K^−1^), b: Temkin constant (L mg^−1^), K_T_: Equilibrium binding constant.

**Figure 3:**
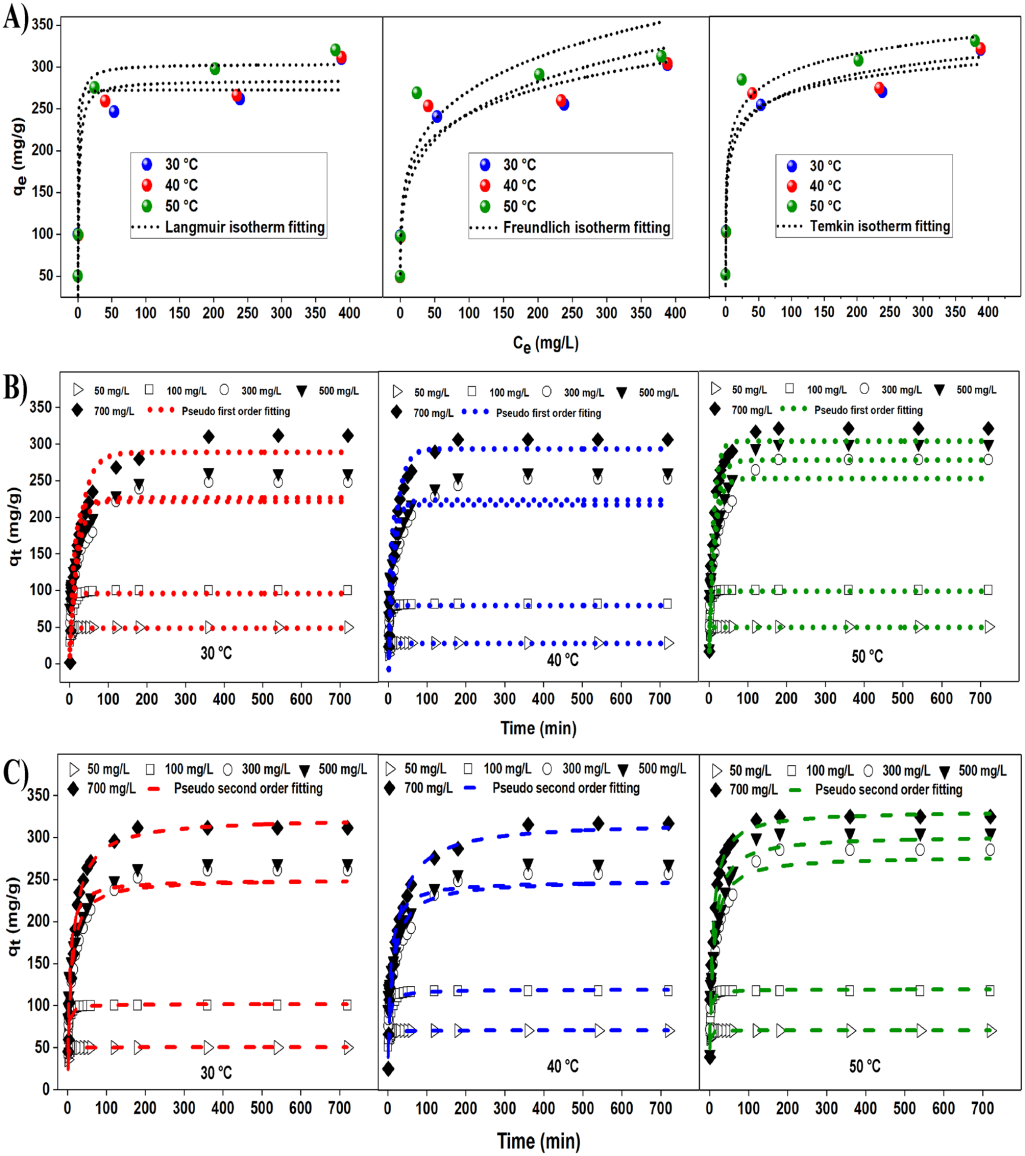
(**A**) Different isotherm models (Langmuir, Freundlich and Temkin), (**B**) Pseudo first order model plots, and (**C**) Pseudo second order model plots for adsorption of MB on activated carbon at different temperatures 30, 40 and 50 °C.

The adsorption isotherm fitting plotted for each isotherm is as shown in Fig. 3A and the evaluated parameters are as shown in Table 5. Based on the results obtained, the experimental data fitted all three isotherm models studied. However, the Langmuir and Temkin isotherm models have r^2^ values more than 0.9 for all three temperatures. This showed that monolayer adsorption was favourable at all temperatures, however, r^2^ values of > 0.9 for Freundlich isotherm obtained at 30 and 40 °C showed that multilayer adsorption may have occurred as well. The value of 1/n for Freundlich isotherm lies from 0 to 1, which shows multilayer adsorption taking place on the heterogeneous surface of AC^51^. However, multilayer adsorption of dye on the AC was not favoured at 50 °C (r^2^ = 0.86). The decrease in the r^2^ value from 0.98 to 0.95 for Temkin model as the temperature increases explains that the heat for adsorption decreases linearly as the adsorbent surface is saturated with adsorbates.

**Table 5:** Parameters for Langmuir, Freundlich and Temkin isotherm models at different temperatures.

Isotherm	Parameters	Temperature (°C)		
		30	40	50
	*q*_*e,exp*_	310.2	311.6	320.4
Langmuir	q_max_	272.6	282.5	302.7
*q*_*m*_K_L_C_e_ (1 + K_L_C_e_)	K_L_ (L mg^−1^)	6.51	0.61	0.86
	r^2^	0.94	0.93	0.95
	χ^2^	5.19	3.00	1.03
Freundlich	*q*_*e,cal*_	313.3	331.0	362.3
	K_F_ (mg g^−1^) (L mg^−1^)^1/n^	115.8	97.6	111.5
	1/n	0.17	0.21	0.20
	r^2^	0.95	0.90	0.86
	χ^2^	0.03	1.14	4.85
Temkin	*q*_*e,cal*_	293.3	301.7	324.6
	K_T_ (L g^−1^)	420.0	68.5	140.7
	b (J mol^−1^)	103.3	88.0	89.9
	r^2^	0.98	0.95	0.95
	*χ*^*2*^	0.97	0.32	0.05

### Adsorption kinetics

The adsorption for MB dye by AC in terms of surface adsorption and diffusion into the internal pores was studied by two kinetic studies, pseudo first and pseudo second order. The non-linearised version of the pseudo first and pseudo second order models are stated below in Eqs. (7) and (8), respectively.

7 $$q_{t} = q_{e} \left( {{1}{-}{\text{e}}^{{{\text{k1}}}} {\text{t}}} \right)$$

8 $$q_{t} = \frac{{{\text{q}}_{{\text{e}}}^{{2}} {\text{k}}_{{2}} {\text{t}}}}{{[{1 } + {\text{q}}_{{\text{t}}} {\text{k}}_{{2}} {\text{t}}]}}$$

where *k*_1_: Equilibrium rate constant for pseudo first order (L min^−1^), *k*_2_: Equilibrium rate constant for pseudo second order (L min^−1^).

The graphs plotted by fitting the experimental data with the kinetic model are as shown in Fig. 3B,C for pseudo first order and pseudo second order, respectively. Table 6 shows the calculated parameters for the two kinetic models. It can be seen that pseudo second order model fits the experimental data better than pseudo first order model with higher r^2^ and lower *χ*^*2*^ values for all the adsorption experiments regardless of the concentrations and the temperatures. This explains that the adsorption between MB dye and parenchyma fibre based AC favours chemisorption over physisorption^52^. A previous study showed that pseudo second order kinetic model was favoured for the adsorption of methylene blue dye by fly ash as the adsorbent surface is primarily occupied by the dye molecules, leading to a competitive adsorption scenario with water due to the surface being covered by water ^53^. The value of rate constant, *k*_*2*,_ decreased with increasing initial dye concentration for all temperatures. This could be due to the competition between the dye molecules at higher initial dye concentrations compared to lower concentrations^54^. The ability to adsorb dye molecules appears to be dependent on the availability of active sites.

**Table 6:** Parameters for pseudo first and pseudo second order kinetics at different temperatures.

T (K)	C_*i*_ (mg g^−1^)	Pseudo 1st order kinetic					Pseudo 2nd order kinetic			
		*q*_*e,exp*_ (mg g^−1^)	*q*_*e,cal*_ (mg g^−1^)	k_1_ (L min^−1^)	χ^2^	r^2^	*q*_*e,cal*_ (mg g^−1^)	k_2_ (L min^−1^)	χ^2^	r^2^
303.15	50	50.0	48.8	0.82	0.03	0.59	50.6	0.048	0.01	0.97
	100	99.9	96.0	0.23	0.16	0.92	101.6	0.007	0.03	0.99
	300	246.3	226.5	0.05	1.73	0.80	250.7	0.0005	0.08	0.92
	500	261.5	221.4	0.09	7.26	0.62	249.3	0.0009	0.60	0.87
	700	310.2	288.8	0.04	1.59	0.94	323.4	0.0003	0.54	0.99
313.15	50	50.0	49.5	1.05	0.01	0.85	50.2	0.03	0.001	0.90
	100	99.1	98.1	0.44	0.01	0.95	101.8	0.003	0.07	0.98
	300	259.4	233.6	0.08	2.85	0.79	240.8	0.0003	1.44	0.91
	500	265.9	227.4	0.18	6.52	0.69	239.0	0.0006	3.03	0.82
	700	311.6	299.5	0.06	0.49	0.97	314.2	0.0002	0.02	0.98
323.15	50	50.0	49.3	1.40	0.01	0.63	50.3	0.07	0.002	0.94
	100	99.4	98.3	0.39	0.01	0.91	102.1	0.007	0.07	0.97
	300	275.4	252.4	0.07	2.10	0.80	270.3	0.0004	0.10	0.93
	500	297.9	277.4	0.07	1.51	0.85	296.5	0.0004	0.01	0.94
	700	320.4	303.5	0.09	0.94	0.95	327.7	0.0004	0.16	0.99

### Adsorption thermodynamics

The values for thermodynamic parameters such as Gibb’s energy (∆G^0^), enthalpy (∆H^0^), and entropy (∆S^0^) were obtained based on equations as follows:

9 $$ln\left( {K_{d} } \right) = \frac{{\Delta {\text{S}}^\circ }}{{\text{R}}} - \frac{{\Delta {\text{H}}^\circ }}{{{\text{RT}}}}$$

10 $$K_{d} = qe/{\text{ Ce}}$$

11 $$\Delta {\text{G}}^\circ = \Delta {\text{H}}^\circ - {\text{ T}}\Delta {\text{S}}^\circ$$

where T: Absolute temperature (K), R: Universal gas constant (8.314 J mol^−1^ K^−1^), *K*_*d*_: Thermodynamic adsorption equilibrium constant, ∆H°: Enthalpy change, ∆S°: Entropy change, ∆G°: Gibbs free energy change.

**Figure 4:**
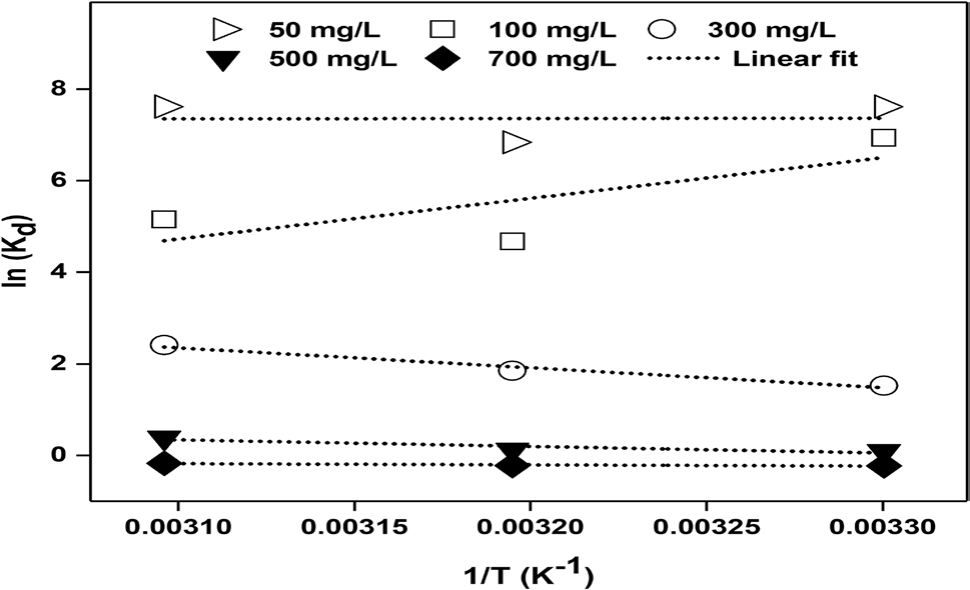
Van’t Hoff plot to determine thermodynamic parameters for different initial dye concentrations and temperatures.

Figure 4 shows the Van’t Hoff plot and Table 7 shows the thermodynamic parameters for this study. The negative values for ∆G^0^ at all temperatures for the initial dye concentrations from 50 to 500 mg L^−1^ show that the adsorption is spontaneous thermodynamically except for 700 mg L^−1^. When the dye concentration reaches a high level, it's likely that the available adsorption sites on the adsorbent surface become fully occupied by dye molecules. Once the surface is saturated in this manner, any additional adsorption becomes less favourable, resulting in a positive ∆G^0^.

**Table 7:** Thermodynamic parameters for different initial dye concentrations at 303.15, 313.15 and 323.15 K.

Initial dye concentration (mg L^−1^)	Thermodynamic parameters				
	∆G^0^ (kJ mol^−1^)			∆H^0^ (kJ mol^−1^)	∆S^0^ (J mol^−1 ^K^−1^)
	303.15 K	313.15 K	323.15 K		
50	− 18,546.0	− 19,136.3	− 19,726.6	− 660.1	59.0
100	− 16,374.4	− 14,478.8	− 12,583.2	− 73,810.9	189.6
300	− 3623.2	− 4936.9	− 6250.5	36,179.2	131.4
500	− 163.8	− 562.9	− 961.9	11,928.1	39.9
700	707.5	657.6	607.8	2219.0	5.0

The ∆H^0^ value for initial dye concentration of 50 and 100 mg L^−1^ favours physisorption whereas for 300, 500 and 700 mg L^−1^ chemisorption might have occurred. It was reported previously that enthalpy values of lower than 40 kJ mol^−1^ indicates physisorption whereas values more than 40 kJ mol^−1^ indicates chemisorption^55–57^. The positive ∆S^0^ values for all the initial dye concentrations indicate randomness and stability in the adsorption process.

## FTIR spectra analysis

**Figure 5:**
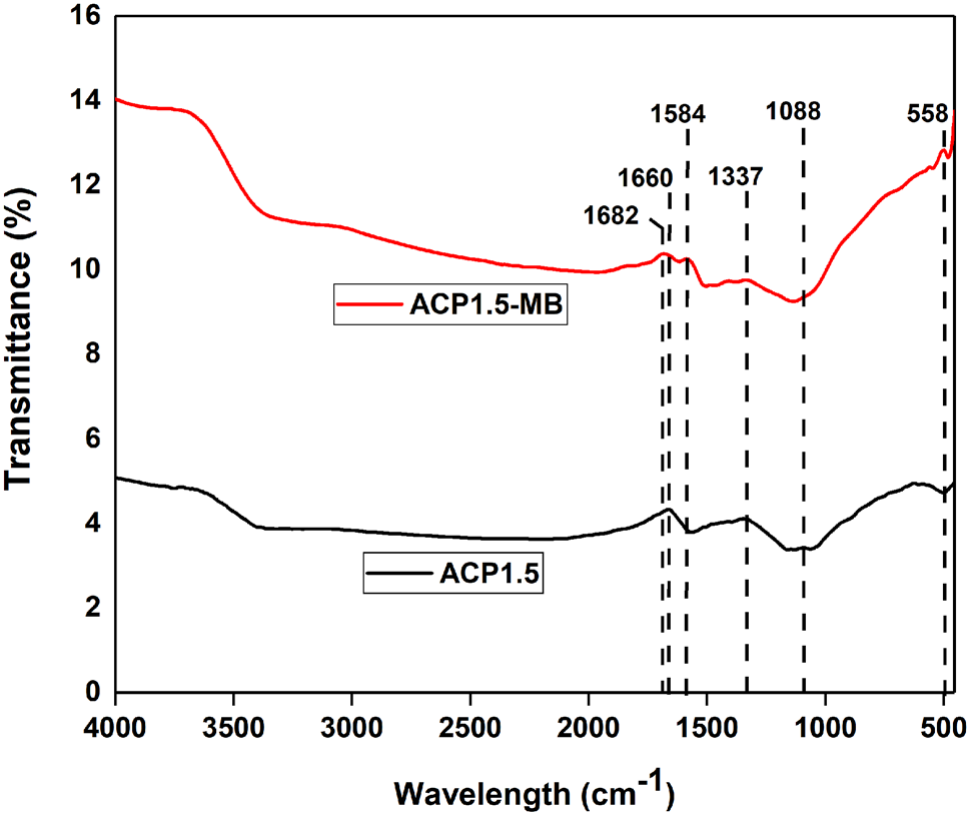
FTIR spectra of parenchyma fibre based activated before (ACP1.5) and after (ACP1.5-MB) adsorption of MB.

The FTIR analysis was carried out for ACP1.5 before and after adsorption of MB as shown in Fig. 5. The FTIR spectrum for the AC before adsorption shows strong peak at wavelength of 1660 cm^−1^. This peak could be due to the stretching of amide I which belongs to protein^58^. It is undeniable that the major source for nitrogen in biomass wood is from proteins and this justifies the availability of nitrogen in parenchyma-based AC. A very slight peak at 1088 cm^−1^ could be due to two different groups such as the PO_4_ which ionised from H_3_PO_4_ ester and the P–O–P group from polyphosphate^59^. These phosphate derivatives originated from this study's chemical treatment conducted with H_3_PO_4_. There is a distinction in FTIR spectra between the surface of the AC before and after adsorption. For instance, the two peaks with wavenumber of 1682 and 1584 cm^−1^ on the AC surface after adsorption may be due to the carboxylic acid (–COOH) and nitromethane group (CH_3_NO_2_), respectively, which were not present for AC before adsorption^60,61^. This shows the presence of MB on the surface of the AC after adsorption. The slight peak for both AC with wavelength of 1337 cm^−1^ is due to the hydroxyl group (–C–OH) in-plane stretching which can be due to the hydrogen bond^62^. The small peak at 558 cm^−1^ may be due to both hydroxyl group out-of-plane bending and alkane group (–C–C) from aromatic hydrogen^63^. The FTIR spectra shows that there is a distinction between the surface of AC before and adsorption of MB dye.

## Elucidation of adsorption mechanism

**Figure 6:**
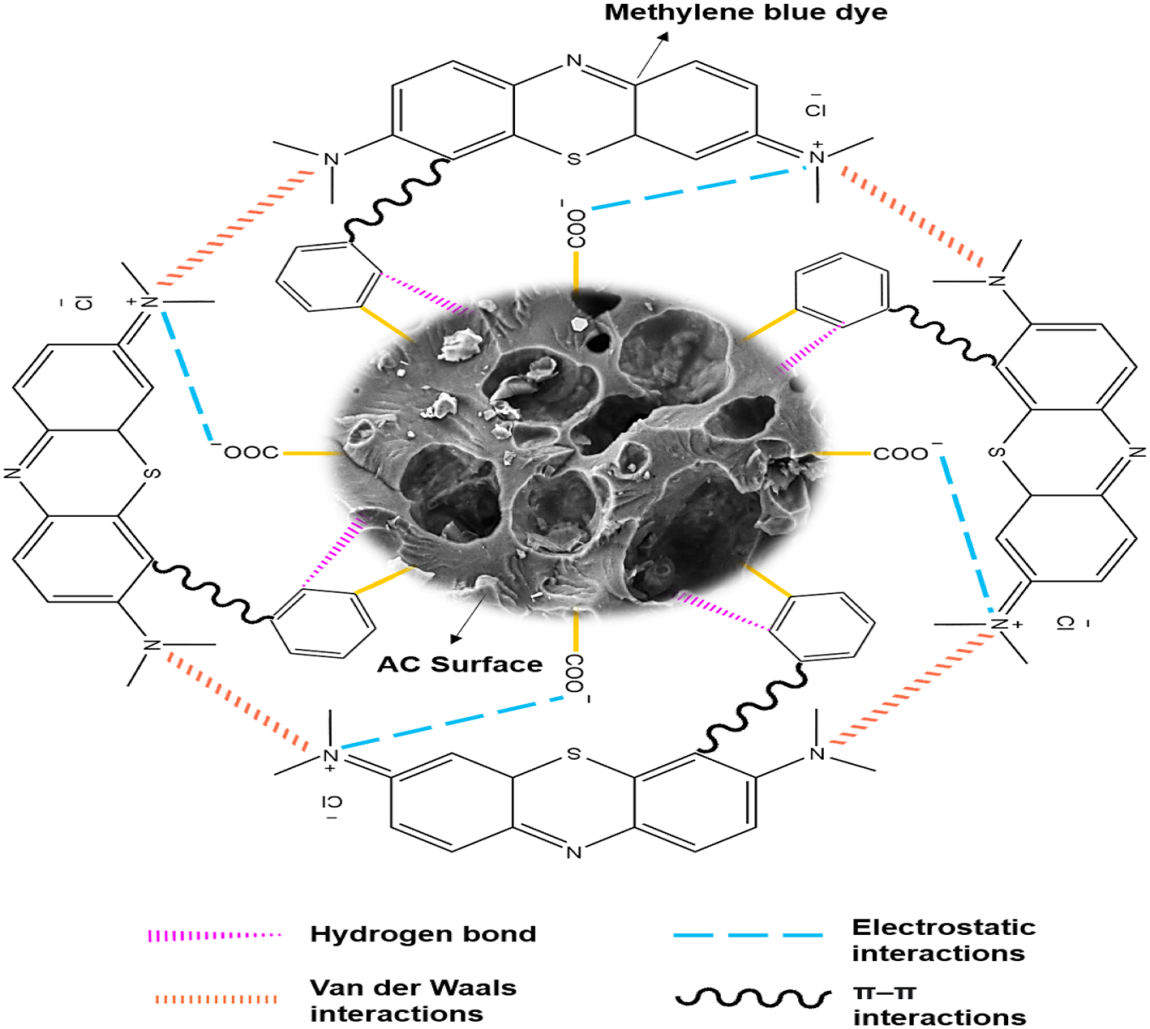
Possible adsorption mechanism involved between MB and AC made from parenchyma fibre (ACP1.5).

A graphical approach to elucidate the adsorption mechanisms between the dye molecules and the AC is shown in Fig. 6. The four possible interactions involved in this study were hydrogen bonding, van der Waals, electrostatic, and π–π interactions. The existence of electrostatic interactions can be explained due to the increase in the adsorption capacity as the pH of the adsorption medium increased from 3 to 11. Electrostatic interactions may occur between the negatively charged carboxylate group from the AC made from lignocellulosic parenchyma fibre and the positively charged N^+^ group in the MB. Another possible interaction is the H-bonding which might have contributed by the OH^−^ ions added during the adjustment of pH. Besides, the π–π interactions is possible between the aromatic ring that exist in the lignin of the parenchyma fibre and the MB. Although H_3_PO_4_ was used to treat the parenchyma fibres initially, it was reported by several literatures that, it is not possible to remove lignin from the lignocellulosic compound efficiently with only H_3_PO_4_^64,65^. Van der Waals’ forces may have also occurred for the adsorption in this study. The weak attraction force between partial electric charges arising from polar molecules and repulsive force will hold molecules in place and form a stable structure^66^. At lower concentration of dye, the dye molecules diffuse to the internal pores of the adsorbent and adsorb. Adsorption increases the concentration of dye molecules on the surface of the adsorbent, which may have created a stable complex and promote the removal of dye from the solution.

## Conclusion

In conclusion, an AC with a large surface area (1599.8 m^2^ g^−1^), pore volume (1.0), and high adsorption capacity (320.4 mg g^−1^) was synthesised from parenchyma fibre from OPT. The adsorption experiments with different parameters show that ACP1.5 has excellent potential in the adsorption of MB dye, one of the abundantly used textile dyes. It was able to remove 78.5% of dye with least amount of AC (12.5 mg). The AC was robust to different ranges of pH, however neutral pH was optimum for the adsorption process. The adsorption kinetic and isotherm models were used to study the effect of initial dye concentration, temperature, time, and stirring speed on the adsorption capacity. The experimental data well fitted the isotherm models in the following order: Temkin (r^2^ = 0.98, 0.95, 0.95) > Langmuir (r^2^ = 0.94, 0.93, 0.95) > Freundlich (r^2^ = 0.95, 0.90, 0.86), and favours chemisorption (pseudo-second order). In addition, the adsorption is endothermic for lower initial dye concentrations (50 and 100 mg L^−1^) and exothermic for higher initial dye concentrations (300, 500, and 700 mg L^−1^). Elucidation of the adsorption mechanism explains the possible mechanisms (hydrogen bonding, van der Waals, electrostatic, and π–π interactions) involved and can be helpful in forecasting a desired application for future applications. This study shows that AC synthesised from OPT fibre can be a potential adsorbent in dye wastewater treatment. However, further improvements can be made by investigating the effect of carbonisation conditions on the adsorption experiments with wastewater, and reusability studies of the synthesised AC.

### Supplementary Information

Supplementary Information.

## Data Availability

All data generated or analysed during this study are included in this published article and its Supplementary information files.

## Abbreviations

AC: Activated carbon
ACP1.5: Activated carbon from parenchyma fibre with acid to precursor ratio of 1.5
ACVB1.5: Activated carbon from vascular bundle fibre with acid to precursor ratio of 1.5
BET: Brunauer–Emmett–Teller
EDX: Energy dispersive X-ray spectroscopy
FTIR: Fourier transform infrared
H_3_PO_4_: Phosphoric acid
IR: Impregnation ratio
MB: Methylene blue
OPT: Oil palm trunk
rpm: Revolution per minute
SEM: Scanning electron microscope

## References

[1] Al-Tohamy R (2022). A critical review on the treatment of dye-containing wastewater: Ecotoxicological and health concerns of textile dyes and possible remediation approaches for environmental safety. Ecotoxicol. Environ. Saf..

[2] Oladoye PO, Ajiboye TO, Omotola EO, Oyewola OJ (2022). Methylene blue dye: Toxicity and potential technologies for elimination from (waste) water. Results Eng..

[3] Din MI, Khalid R, Najeeb J, Hussain Z (2021). Fundamentals and photocatalysis of methylene blue dye using various nanocatalytic assemblies-a critical review. J. Clean. Prod..

[4] El-Sayed GO, Yehia MM, Asaad AA (2014). Assessment of activated carbon prepared from corncob by chemical activation with phosphoric acid. Water Resour. Ind..

[5] Guo Y (2020). Porous activated carbons derived from waste sugarcane bagasse for CO2 adsorption. Chem. Eng. J..

[6] Hameed BH, Din ATM, Ahmad AL (2007). Adsorption of methylene blue onto bamboo-based activated carbon: Kinetics and equilibrium studies. J. Hazard. Mater..

[7] Feng P, Li J, Wang H, Xu Z (2020). Biomass-based activated carbon and activators: Preparation of activated carbon from corncob by chemical activation with biomass pyrolysis liquids. ACS Omega.

[8] Al-Sarraf MK, Abbas M (2020). Preparation of activated carbon from sunflower seeds. Plant Arch..

[9] Akl MA, Dawy MB, Serage AA (2014). Efficient removal of phenol from water samples using sugarcane bagasse based activated carbon. J. Anal. Bioanal. Tech..

[10] Williams PT, Reed AR (2006). Development of activated carbon pore structure via physical and chemical activation of biomass fibre waste. Biomass Bioenergy.

[11] Altıntıg E (2021). Facile synthesis of zinc oxide nanoparticles loaded activated carbon as an eco-friendly adsorbent for ultra-removal of malachite green from water. Environ. Technol. Innov..

[12] Jawad AH (2022). Mesoporous activated carbon from mangosteen (*Garcinia mangostana*) peels by H3PO4 assisted microwave: Optimization, characterization, and adsorption mechanism for methylene blue dye removal. Diam. Relat. Mater..

[13] Altıntıg E, Altundag H, Tuzen M, Sarı A (2017). Effective removal of methylene blue from aqueous solutions using magnetic loaded activated carbon as novel adsorbent. Chem. Eng. Res. Des..

[14] Muda K, Ezechi EH (2019). Overview of trends in crude palm oil production and economic impact in Malaysia. Sriwij. J. Environ..

[15] Oseghale SD, Mohamed AF, Chikere AO (2017). Status evaluation of palm oil waste management sustainability in Malaysia. OIDA Int. J. Sustain. Dev..

[16] Pulingam T (2022). Oil palm trunk waste: Environmental impacts and management strategies. Ind. Crops Prod..

[17] Hashim R (2011). Characterization of raw materials and manufactured binderless particleboard from oil palm biomass. Mater. Des..

[18] Darwis A (2013). Vascular bundle distribution effect on density and mechanical properties of oil palm trunk. Asian J. Plant Sci..

[19] Lim A (2020). Synthesis, characterization, adsorption isotherm, and kinetic study of oil palm trunk-derived activated carbon for tannin removal from aqueous solution. ACS Omega.

[20] Li Y (2013). Comparative study of methylene blue dye adsorption onto activated carbon, graphene oxide, and carbon nanotubes. Chem. Eng. Res. Des..

[21] Ali R, Aslam Z, Shawabkeh RA, Asghar A, Hussein IA (2020). BET, FTIR, and RAMAN characterizations of activated carbon from waste oil fly ash. Turk. J. Chem..

[22] Du H (2020). Red dye extracted sappan wood waste derived activated carbons characterization and dye adsorption properties. Diam. Relat. Mater..

[23] Zakaria R, Jamalluddin NA, Abu Bakar MZ (2021). Effect of impregnation ratio and activation temperature on the yield and adsorption performance of mangrove based activated carbon for methylene blue removal. Results Mater..

[24] Liu Q-S, Zheng T, Wang P, Guo L (2010). Preparation and characterization of activated carbon from bamboo by microwave-induced phosphoric acid activation. Ind. Crops Prod..

[25] Kang S (2018). Valorization of humins by phosphoric acid activation for activated carbon production. Biomass Convers. Biorefinery.

[26] Han Q, Wang J, Goodman BA, Xie J, Liu Z (2020). High adsorption of methylene blue by activated carbon prepared from phosphoric acid treated eucalyptus residue. Powder Technol..

[27] Shamsuddin MS, Yusoff NRN, Sulaiman MA (2016). Synthesis and characterization of activated carbon produced from kenaf core fiber using H3PO4 activation. Proced. Chem..

[28] Igbokwe JT (2012). Adsorption performance of packed bed column for the removal of lead (ii) using oil palm fibre. Int. J. Appl. Sci. Technol..

[29] Yorgun S, Yıldız D, Şimşek YE (2016). Activated carbon from paulownia wood: Yields of chemical activation stages. Energy Sources Part A Recover. Util. Environ. Eff..

[30] Yakout SM, El-Deen GS (2016). Characterization of activated carbon prepared by phosphoric acid activation of olive stones. Arab. J. Chem..

[31] Fierro V, Torné-Fernández V, Celzard A (2006). Kraft lignin as a precursor for microporous activated carbons prepared by impregnation with ortho-phosphoric acid: Synthesis and textural characterisation. Microporous Mesoporous Mater..

[32] Davamani V (2022). Phytolith-occluded carbon sequestration potential of oil palm plantation in Tamil Nadu. ACS Omega.

[33] Mojoudi N (2019). Phenol adsorption on high microporous activated carbons prepared from oily sludge: Equilibrium, kinetic and thermodynamic studies. Sci. Rep..

[34] Mi T, Chen L, Xin S, Yu X (2015). Activated carbon from the Chinese herbal medicine waste by H3PO4 activation. J. Nanomater..

[35] Liu X-J, Li M-F, Singh SK (2021). Manganese-modified lignin biochar as adsorbent for removal of methylene blue. J. Mater. Res. Technol..

[36] Kassimi AE, Achour Y, Himri ME, Laamari MR, Haddad ME (2021). High efficiency of natural Safiot Clay to remove industrial dyes from aqueous media: Kinetic, isotherm adsorption and thermodynamic studies. Biointerface Res. Appl. Chem.

[37] Idan IJ, Abdullah LC, Choong TSY, Jamil SNABM (2018). Equilibrium, kinetics and thermodynamic adsorption studies of acid dyes on adsorbent developed from kenaf core fiber. Adsorpt. Sci. Technol..

[38] Jawad AH, Mubarak NSA, Abdulhameed AS (2020). Tunable Schiff’s base-cross-linked chitosan composite for the removal of reactive red 120 dye: Adsorption and mechanism study. Int. J. Biol. Macromol..

[39] Albroomi HI, Elsayed MA, Baraka A, Abdelmaged MA (2017). Batch and fixed-bed adsorption of tartrazine azo-dye onto activated carbon prepared from apricot stones. Appl. Water Sci..

[40] Banerjee S, Chattopadhyaya MC (2017). Adsorption characteristics for the removal of a toxic dye, tartrazine from aqueous solutions by a low cost agricultural by-product. Arab. J. Chem..

[41] Hamzezadeh A, Rashtbari Y, Afshin S, Morovati M, Vosoughi M (2022). Application of low-cost material for adsorption of dye from aqueous solution. Int. J. Environ. Anal. Chem..

[42] Al-Ghouti MA, Al-Absi RS (2020). Mechanistic understanding of the adsorption and thermodynamic aspects of cationic methylene blue dye onto cellulosic olive stones biomass from wastewater. Sci. Rep..

[43] de Yuso AM, Rubio B, Izquierdo MT (2014). Influence of activation atmosphere used in the chemical activation of almond shell on the characteristics and adsorption performance of activated carbons. Fuel Process. Technol..

[44] Jawad AH, Malek NNA, Khadiran T, Alothman ZA, Yaseen ZM (2022). Mesoporous high-surface-area activated carbon from biomass waste via microwave-assisted-H3PO4 activation for methylene blue dye adsorption: An optimized process. Diam. Relat. Mater..

[45] Jawad AH (2021). Microporous activated carbon developed from KOH activated biomass waste: Surface mechanistic study of methylene blue dye adsorption. Water Sci. Technol..

[46] Yusop MFM, Ahmad MA, Rosli NA, Abd Manaf ME (2021). Adsorption of cationic methylene blue dye using microwave-assisted activated carbon derived from acacia wood: Optimization and batch studies. Arab. J. Chem..

[47] Abdulhameed AS (2021). Statistical modeling and mechanistic pathway for methylene blue dye removal by high surface area and mesoporous grass-based activated carbon using K2CO3 activator. J. Environ. Chem. Eng..

[48] Zhou Q-Q, Qiu L, Zhu M-Q (2022). Eucommia ulmoides Oliver derived magnetic activated carbon for eliminating methylene blue from dyeing wastewater and its economic efficiency assessment. Ind. Crops Prod..

[49] Maia LS (2021). Activated carbon from palm fibres used as an adsorbent for methylene blue removal. J. Polym. Environ..

[50] Baloo L (2021). Adsorptive removal of methylene blue and acid orange 10 dyes from aqueous solutions using oil palm wastes-derived activated carbons. Alex. Eng. J..

[51] Zhang Z, Zhao X, Jv X, Lu H, Zhu L (2017). A simplified method for synthesis of l-tyrosine modified magnetite nanoparticles and its application for the removal of organic dyes. J. Chem. Eng. Data.

[52] Wang Y (2017). Removal of Pb (II) and methylene blue from aqueous solution by magnetic hydroxyapatite-immobilized oxidized multi-walled carbon nanotubes. J. Colloid Interface Sci..

[53] Karaca H, Altıntığ E, Türker D, Teker M (2018). An evaluation of coal fly ash as an adsorbent for the removal of methylene blue from aqueous solutions: Kinetic and thermodynamic studies. J. Dispers. Sci. Technol..

[54] Saxena M, Sharma N, Saxena R (2020). Highly efficient and rapid removal of a toxic dye: Adsorption kinetics, isotherm, and mechanism studies on functionalized multiwalled carbon nanotubes. Surf. Interfaces.

[55] Işık B, Uğraşkan V (2021). Adsorption of methylene blue on sodium alginate–flax seed ash beads: Isotherm, kinetic and thermodynamic studies. Int. J. Biol. Macromol..

[56] Argun ME (2008). Use of clinoptilolite for the removal of nickel ions from water: Kinetics and thermodynamics. J. Hazard. Mater..

[57] Nollet H, Roels M, Lutgen P, Van der Meeren P, Verstraete W (2003). Removal of PCBs from wastewater using fly ash. Chemosphere.

[58] Marzec KM (2014). Visualization of the biochemical markers of atherosclerotic plaque with the use of Raman, IR and AFM. J. Biophoton..

[59] Kazmierczak-Razna J, Nowicki P, Wiśniewska M, Nosal-Wiercińska A, Pietrzak R (2017). Thermal and physicochemical properties of phosphorus-containing activated carbons obtained from biomass. J. Taiwan Inst. Chem. Eng..

[60] Wang P (2019). Poly-allylamine hydrochloride and fucoidan-based self-assembled polyelectrolyte complex nanoparticles for cancer therapeutics. J. Biomed. Mater. Res. Part A.

[61] Pal C, Hazra A, Ghosh PN, Kshirsagar RJ (1997). High resolution Fourier transform infrared spectrum and vibration-rotation analysis of the B-type 1584 cm− band of nitromethane. J. Mol. Struct..

[62] Chen Q (2023). Quantitative measurements of DP in cellulose paper based on terahertz spectroscopy. Polymers (Basel).

[63] Yang H, Yan R, Chen H, Lee DH, Zheng C (2007). Characteristics of hemicellulose, cellulose and lignin pyrolysis. Fuel.

[64] Wang Q (2014). Pretreating lignocellulosic biomass by the concentrated phosphoric acid plus hydrogen peroxide (PHP) for enzymatic hydrolysis: Evaluating the pretreatment flexibility on feedstocks and particle sizes. Bioresour. Technol..

[65] Zhang YP (2007). Fractionating recalcitrant lignocellulose at modest reaction conditions. Biotechnol. Bioeng..

[66] Ketema A, Worku A (2020). Review on intermolecular forces between dyes used for polyester dyeing and polyester fiber. J. Chem..

