# Supplementary material for: Sustainable oil palm trunk fibre based activated carbon for the adsorption of methylene blue
Source: Sci Rep. 2023 Dec 13;13:22137. doi: 10.1038/s41598-023-49079-0 (PMC10719241; doi:10.1038/s41598-023-49079-0)
Supplement: Supplementary file 1 — Supplementary Information. [file 41598_2023_49079_MOESM1_ESM.docx]

**Raw data**

**Effect of dosage of AC**

| Amount of adsorbent | Percentage of dye removal (%) | Standard deviation | Percentage of dye removal (%) | Standard deviation |
| --- | --- | --- | --- | --- |
|  | ACP1.5 | | ACVB1.5 | |
| 12.5 | 78.54 | 1.2 | 59.97 | 1.5 |
| 25 | 96.1 | 1 | 90.76 | 1.4 |
| 50 | 97.41 | 1.5 | 97.36 | 1.8 |

#### Effect of pH of the solution

| pH | Adsorption capacity (mg/g) | Standard deviation |
| --- | --- | --- |
| ACP1.5 | | |
| 3 | 92.70057 | 1.2 |
| 5 | 92.70057 | 1.6 |
| 7 | 95.82219 | 0.9 |
| 9 | 93.13706 | 0.8 |
| 11 | 99.33522 | 1 |

#### Effect of initial dye concentration and contact time

| Time (min) | Adsorption (mg/g) | | | | |
| --- | --- | --- | --- | --- | --- |
|  | 50 (mg/L) | 100 (mg/L) | 300 (mg/L) | 500 (mg/L) | 700 (mg/L) |
| 1 | 34.844 | 30.282 | 55.686 | 76.725 | 1.7359 |
| 2 | 37.899 | 39.012 | 64.416 | 94.185 | 1.7359 |
| 3 | 38.772 | 57.781 | 73.146 | 102.91 | 45.385 |
| 4 | 42.264 | 57.781 | 86.241 | 105.82 | 89.034 |
| 5 | 41.391 | 58.945 | 86.241 | 108.73 | 103.58 |
| 10 | 47.895 | 73.058 | 100.79 | 126.19 | 118.13 |
| 15 | 49.612 | 82.225 | 121.16 | 137.83 | 132.68 |
| 20 | 49.859 | 87.899 | 135.71 | 149.47 | 161.78 |
| 25 | 49.932 | 92.264 | 144.44 | 164.02 | 176.33 |
| 30 | 49.975 | 96.323 | 156.08 | 171.3 | 190.88 |
| 40 | 49.99 | 97.284 | 164.81 | 182.94 | 205.43 |
| 50 | 49.99 | 98.331 | 172.08 | 190.21 | 219.98 |
| 60 | 49.99 | 99.015 | 179.36 | 198.94 | 234.53 |
| 120 | 49.99 | 99.903 | 221.55 | 229.5 | 268 |
| 180 | 49.99 | 99.99 | 239.01 | 246.96 | 279.63 |
| 360 | 49.99 | 99.99 | 246.29 | 261.51 | 310.19 |
| 540 | 49.99 | 99.99 | 247.74 | 261.51 | 311.64 |
| 720 | 49.99 | 10 | 99.99 | 7 | 247.74 |

**Effect of temperature and initial dye concentration**

| Initial dye (mg/L) | Adsorption capacity (mg/g) | | |
| --- | --- | --- | --- |
|  | 30 °C | 40 °C | 50 °C |
| 50 | 49.97541 | 49.94631 | 49.97541 |
| 100 | 99.90266 | 99.07333 | 99.42252 |
| 300 | 246.28707 | 259.38178 | 275.38644 |
| 500 | 261.50604 | 265.87094 | 297.88026 |
| 700 | 310.18929 | 311.64426 | 320.37407 |

**Experimental data for nonlinear isotherm model fitting**

| Initial dye (mg/L) | Equilibrium concentration (mg/L) | | | Equilibrium adsorption capacity (mg/g) | | |
| --- | --- | --- | --- | --- | --- | --- |
|  | 30 °C | 40 °C | 50 °C | 30 °C | 40 °C | 50 °C |
| 50 | 0.0246 | 0.0537 | 0.02459 | 49.97541 | 49.946 | 49.97541 |
| 100 | 0.0973 | 0.9267 | 0.57748 | 99.90266 | 99.073 | 99.42252 |
| 300 | 53.713 | 40.618 | 24.61356 | 246.28707 | 259.38 | 275.38644 |
| 500 | 238.49 | 234.13 | 202.11974 | 261.51 | 265.87 | 297.88026 |
| 700 | 388.36 | 388.36 | 379.62593 | 310.19 | 311.64 | 320.37407 |

**Experimental data for nonlinear kinetic model fitting**

1. **30 °C**

| Time (min) | q_t_ (mg/g) | | | | |
| --- | --- | --- | --- | --- | --- |
|  | 50 mg/L | 100 mg/L | 300 mg/L | 500 mg/L | 700 mg/L |
| 1 | 34.844 | 30.282 | 55.686 | 76.725 | 1.7359 |
| 2 | 37.899 | 39.012 | 64.416 | 94.185 | 1.7359 |
| 3 | 38.772 | 57.781 | 73.146 | 102.91 | 45.385 |
| 4 | 42.264 | 57.781 | 86.241 | 105.82 | 89.034 |
| 5 | 41.391 | 58.945 | 86.241 | 108.73 | 103.58 |
| 10 | 47.895 | 73.058 | 100.79 | 126.19 | 118.13 |
| 15 | 49.612 | 82.225 | 121.16 | 137.83 | 132.68 |
| 20 | 49.859 | 87.899 | 135.71 | 149.47 | 161.78 |
| 25 | 49.932 | 92.264 | 144.44 | 164.02 | 176.33 |
| 30 | 49.99 | 96.323 | 156.08 | 171.3 | 190.88 |
| 40 | 49.99 | 97.284 | 164.81 | 182.94 | 205.43 |
| 50 | 49.99 | 98.331 | 172.08 | 190.21 | 219.98 |
| 60 | 49.99 | 99.015 | 179.36 | 198.94 | 234.53 |
| 120 | 49.99 | 99.859 | 221.55 | 229.5 | 268 |
| 180 | 49.99 | 99.99 | 239.01 | 246.96 | 279.63 |
| 360 | 49.99 | 99.99 | 247.74 | 261.51 | 310.19 |
| 540 | 49.99 | 99.99 | 247.74 | 260.05 | 311.64 |
| 720 | 49.99 | 99.99 | 247.74 | 260.05 | 311.64 |

1. **40 °C**

| Time (min) | q_t_ (mg/g) | | | | |
| --- | --- | --- | --- | --- | --- |
|  | 50 mg/L | 100 mg/L | 300 mg/L | 500 mg/L | 700 mg/L |
| 1 | 35.717 | 43.377 | 64.416 | 85.455 | 45.385 |
| 2 | 40.227 | 59.964 | 77.511 | 98.55 | 45.385 |
| 3 | 45.029 | 70.585 | 83.331 | 111.64 | 59.935 |
| 4 | 46.178 | 76.987 | 103.7 | 134.92 | 89.034 |
| 5 | 48.229 | 80.624 | 105.16 | 133.47 | 103.58 |
| 10 | 49.597 | 90.082 | 128.43 | 152.38 | 132.68 |
| 15 | 49.932 | 94.738 | 142.98 | 171.3 | 161.78 |
| 20 | 49.99 | 97.444 | 160.44 | 175.66 | 190.88 |
| 25 | 49.99 | 98.477 | 169.17 | 190.21 | 219.98 |
| 30 | 49.99 | 99.088 | 177.9 | 193.12 | 234.53 |
| 40 | 49.99 | 99.495 | 192.45 | 206.22 | 249.08 |
| 50 | 49.99 | 99.743 | 205.55 | 216.4 | 263.63 |
| 60 | 49.99 | 99.757 | 214.28 | 228.04 | 270.91 |
| 120 | 49.99 | 100 | 237.56 | 248.41 | 295.64 |
| 180 | 49.99 | 100.08 | 252.11 | 262.96 | 311.64 |
| 360 | 49.99 | 100.08 | 260.84 | 268.78 | 311.64 |
| 540 | 49.99 | 100.08 | 260.84 | 268.78 | 311.64 |
| 720 | 49.99 | 100.08 | 260.84 | 268.78 | 311.64 |

1. **50 °C**

| Time (min) | q_t_ (mg/g) | | | | |
| --- | --- | --- | --- | --- | --- |
|  | 50 mg/L | 100 mg/L | 300 mg/L | 500 mg/L | 700 mg/L |
| 1 | 40.082 | 49.197 | 51.321 | 19.981 | 16.286 |
| 2 | 41.973 | 53.707 | 78.966 | 94.185 | 89.034 |
| 3 | 44.592 | 65.493 | 89.15 | 108.73 | 89.034 |
| 4 | 46.964 | 73.786 | 112.43 | 111.64 | 132.68 |
| 5 | 47.997 | 78.005 | 118.25 | 117.46 | 132.68 |
| 10 | 49.655 | 90.373 | 134.25 | 143.65 | 161.78 |
| 15 | 49.917 | 95.174 | 150.26 | 171.3 | 205.43 |
| 20 | 49.99 | 97.735 | 166.26 | 182.94 | 234.53 |
| 25 | 49.99 | 98.797 | 180.81 | 193.12 | 249.08 |
| 30 | 49.99 | 99.423 | 191 | 203.31 | 263.63 |
| 40 | 49.99 | 99.772 | 204.09 | 225.13 | 275.27 |
| 50 | 49.99 | 99.917 | 212.82 | 241.14 | 284 |
| 60 | 49.99 | 99.975 | 221.55 | 251.32 | 289.82 |
| 120 | 49.99 | 99.99 | 263.75 | 293.52 | 316.01 |
| 180 | 49.99 | 99.99 | 278.3 | 299.34 | 320.37 |
| 360 | 49.99 | 99.99 | 278.3 | 299.34 | 320.37 |
| 540 | 49.99 | 99.99 | 278.3 | 299.34 | 320.37 |
| 720 | 49.99 | 99.99 | 278.3 | 299.34 | 320.37 |
